# Identifying Rare Genetic Variants of Immune Mediators as Risk Factors for Autism Spectrum Disorder

**DOI:** 10.3390/genes13061098

**Published:** 2022-06-20

**Authors:** Chunquan Cai, Zhaoqing Yin, Aiping Liu, Hui Wang, Shujuan Zeng, Zhangxing Wang, Huixian Qiu, Shijun Li, Jiaxiu Zhou, Mingbang Wang

**Affiliations:** 1 Tianjin Pediatric Research Institute, Tianjin Key Laboratory of Birth Defects for Prevention and Treatment, Tianjin Children’s Hospital (Children’s Hospital of Tianjin University), No. 238 Longyan Road, Beichen District, Tianjin 300134, China; cqcns6@126.com; 2 Division of Neonatology, The People’s Hospital of Dehong Autonomous Prefecture, Mangshi 678400, China; zhaoqingyin99@sina.com; 3 The Department of Laboratory, Public Health Service Center of Bao’an District, Bao’an District, Shenzhen 518018, China; szchengba@foxmail.com; 4 Xiamen Branch of Children’s Hospital of Fudan University (Xiamen Children’s Hospital), Xiamen 361006, China; 15545257307@163.com; 5 Division of Neonatology, Longgang Central Hospital of Shenzhen, Shenzhen 518116, China; zengshujuansz@163.com (S.Z.); szlgqhx@126.com (H.Q.); 6 Division of Neonatology, Shenzhen Longhua People’s Hospital, Shenzhen 518109, China; wzx776@163.com; 7 Department of Radiology, Chinese People’s Liberation Army General Hospital, Beijing 100853, China; 8 Division of Psychology, Shenzhen Children’s Hospital, Shenzhen 518038, China; 9 Microbiome Therapy Center, South China Hospital of Shenzhen University, Shenzhen 518111, China; 10 Shanghai Key Laboratory of Birth Defects, Division of Neonatology, Children’s Hospital of Fudan University, Shanghai 201102, China

**Keywords:** autism spectrum disorder, immune mediators, HLA-B, HLA-DQB1, LILRB2

## Abstract

Autism spectrum disorder (ASD) affects more than 1% of children, and there is no viable pharmacotherapeutic agent to treat the core symptoms of ASD. Studies have shown that children with ASD show changes in their levels of immune response molecules. Our previous studies have shown that ASD is more common in children with folate receptor autoantibodies. We also found that children with ASD have abnormal gut immune function, which was characterized by a significant increase in the content of immunoglobulin A and an increase in gut-microbiota-associated epitope diversity. These studies suggest that the immune mechanism plays an important role in the occurrence of ASD. The present study aims to systematically assess gene mutations in immune mediators in patients with ASD. We collected genetic samples from 72 children with ASD (2–12 years old) and 107 healthy controls without ASD (20–78 years old). We used our previously-designed immune gene panel, which can capture cytokine and receptor genes, the coding regions of MHC genes, and genes of innate immunity. Target region sequencing (500×) and bioinformatics analytical methods were used to identify variants in immune response genes associated with patients with ASD. A total of 4 rare variants were found to be associated with ASD, including HLA-B: p.A93G, HLA-DQB1: p.S229N, LILRB2: p.R322H, and LILRB2: c.956-4C>T. These variants were present in 44.44% (32/72) of the ASD patients and were detected in 3.74% (4/107) of the healthy controls. We expect these genetic variants will serve as new targets for the clinical genetic assessment of ASD, and our findings suggest that immune abnormalities in children with ASD may have a genetic basis.

## 1. Introduction

Autism spectrum disorders (ASD) are a group of neurodevelopmental disorders which affect around 1% of school-age children and which impose severe economic and social burdens on ordinary families and society [1,2,3,4]. Currently, there is no pharmacotherapeutic agent to treat the core symptoms of ASD [5]. Genetic factors play an important role in autism, and we have previously applied whole-genome sequencing to autism genetic studies and found autism-related mutations [6,7,8]; However, like the clinical heterogeneity of autism, autism also has a complex genetic structure, and large-scale ASD genomic studies have identified a large number of clinically relevant ASD variants; these variants involve common and rare variants in more than 100 risk genes, from point mutations to copy number variations, either inherited or de novo [9,10,11,12,13]. Recent studies have suggested an association of MHC regional variation with ASD and neuropsychiatric diseases [14,15]. Our previous study showed that 77.5% (31/40) of children with ASD were found to be positive for folate receptor autoantibodies (FRAA) [16]; this is consistent with the results of Frye et al. [17,18]. Furthermore, Frye et al. found that exposure to FRAA in pregnant rats was associated with behavioral deficits in offspring [19]; they also found that folinic acid was associated with improvements in core and related symptoms of ASD [20,21]. A number of maternal autoantibodies that can serve as biomarkers for ASD have been identified [22,23,24,25]; meanwhile, animal studies have confirmed the correlation between continuous exposure to ASD-specific maternal autoantibodies throughout gestation in mice and ASD-like behavior in offspring [26,27]. In short, these suggest a possible correlation between autoimmune problems and the development of ASD, and it also suggests the need for systematic research on the MHC region of ASD patients. Considering the importance of the MHC region in mediating host immune responses and its inherent high mutagenicity, high-throughput sequencing of the MHC region is necessary. Cytokines have an important role in neurodevelopment [28,29], and studies have demonstrated that peripheral cytokine profiles at birth are associated with ASD in later childhood [30,31,32,33]. Maternal immune activation (MIA) is an environmental risk factor for ASD, and MIA-mouse offspring often exhibit ASD-like behavior. These mice are widely used as a model of ASD [34]. Choi et al. [35] found that MIA-induced, ASD-like behavior is dependent on maternal IL-17A and that this behavioral abnormality can be improved by blocking IL-17A. There is increasing evidence that serum cytokine abnormalities are present in patients with ASD [36]. Tsilioni et al. found that elevated serum IL-6 and TNF can define the ASD subgroup, a group that benefits from treatment with the natural flavonoid luteolin [37]. A cross-sectional study by Al-Ayadhi of 45 children, aged 6–12 years, with ASD showed that the serum levels of IL-17A were positively correlated with the severity of autism, and nearly 50% of children with autism had elevated serum IL-17A levels. Of these, 67.9% had severe ASD, and 17% had mild-to-moderate ASD [38]. The imbalance of the immune system in patients with ASD was also characterized by an abnormal lymphocyte count, the presence of serum brain-specific autoantibodies, and intestinal immune dysfunction [39]. Imbalanced immune responses in children with ASD may be affected by changes in gut microbiota [40,41], and a large number of studies on ASD gut microbiota have been published [42,43,44,45,46,47]; our previous studies have shown that abnormal intestinal or oral IgA is a potential biomarker for ASD [48,49,50], and that abnormal gut microbiota structure, especially an epitope-associated gut microbiota structure, is associated with increased IgA [51]. In short, these studies suggest an abnormality in immune function in ASD. The aim of this study was to systematically assess gene mutations in immune mediators of children with ASD to determine if there are immune-related genetic defects in children with ASD.

## 2. Method

### 2.1. Study Participants

A total of 72 patients with ASD, 54 males (2–8 years old) and eighteen females (2–11 years old) were recruited from the Department of Psychology at Shenzhen Children’s Hospital. The specific process was as described in our previous study [50]. In short, inclusion criteria were as follows: the patient must be age <14 years, must meet the diagnosis of ASD, and must be classified as “requiring very substantial support” according to the 5th edition of the *Diagnostic and Statistical Manual of Mental Disorders*. Furthermore, inclusion was not limited by the sex of the patient. The exclusion criteria were as follow: The patient must not (1) be suffering from other mental illnesses (such as obsessive–compulsive disorder or ADHD); (2) be suffering from other neurodevelopmental disorders; (3) be suffering from genetic metabolic diseases; (4) be suffering from serious neurological diseases or have a history of brain injury or other major physical diseases; (5) be acutely physically ill. Healthy controls totaled 107 individuals, 57 males (20–78 years old) and 50 females (24–73 years old), from a previously published hepatitis B virus (HBV) cohort study. All healthy controls were non-ASD individuals—adults who were not diagnosed with ASD in childhood as determined by medical history examination, and the inclusion and exclusion criteria are detailed in previously published articles [52,53]. All enrolled individuals were informed of the study, and the enrolled individuals signed informed consent forms. The study was approved by the Ethics Committee of Shenzhen Children’s Hospital.

### 2.2. DNA Extraction and Quality Control

A volume of 3 to 5 mL of whole blood was drawn and stored at −80 °C. Then, genomic DNA was extracted from these whole-blood samples using a PureLink™ Genomic DNA Mini Kit (Thermo Fisher, Foster City, CA, USA). The genomic DNA samples were quantified and controlled for quality using NanoDrop ND2000 (Thermo Fisher). The total amount of DNA was required to be above 1 µg; the purity ratio of A260/280 was required to be in the range of 1.8–2.0; the main DNA band had to be clearly visible; and the fragment size had to appear around 23 K Da, as measured by 0.3% agarose gel electrophoresis.

### 2.3. Library Construction

A DNA sample of 1 µg was fragmented into 150–250 bp-sized fragments using a Bioruptor interrupter (Diagenode, Seraing, Belgium); then, the fragmented DNA was subjected to end-filling, 5′ segment phosphate group repair, and 3′ segment plus A (Enzymatics Inc., Beverly, MA, USA) using an ABI 2720 type of PCR instrument (Thermo Fisher, Hudson, NH, USA). Finally, a synthetic paired-end adapter (Thermo Fisher) suitable for the Illumina HiSeq sequencer (Illumina, San Diego, CA, USA) was ligated using an ABI 2720 PCR machine (Thermo Fisher), and the ligation product was purified using MagPure A3 XP beads (Magen, Guangzhou, China). For the purified ligation product, PCR pre-amplification (KAPA Biosystems, USA) was performed using an ABI 2720 PCR machine (Thermo Fisher), and a synthetic index sequence (Thermo Fisher) capable of distinguishing individual samples was introduced. The parameters were as follows: 95 °C for 4 min, 98 °C for 20 s, 65 °C for 30 s, 5 cycles, 72 °C for 30 s, 72 °C for 5 min, and 12 °C hold. A small-fragment sequencing library was obtained, and 1 µL of the small-fragment library was subjected to quantification using a Qubit dsDNA HS Assay Kit (Thermo Fisher). The concentration of the capture library was determined, and the standard of the qualified library was measured to be greater than 3 ng/µL.

### 2.4. Targeted Region Sequencing

The panel, which targeted the coding region of 404 immune-response molecule genes, included genes related to cytokines, receptors, innate immunity, and adaptive immunity and was designed in our previous study [52]; target region sequencing of the 404 immune-response genes was performed according to our published studies [54,55]. In brief, the small-fragment library was mixed with a hybridization block (iGeneTech, Beijing, China). Then, commercially acquired TargetSeq hybridization buffer (iGeneTech) was thawed at room temperature, mixed, and placed in a 65 °C water bath to preheat until the solution was completely dissolved. For each sample, 20 µL of the hybridization buffer was placed in a PCR tube and incubated in a water bath at 65 °C. Before hybridization, 5 µL of RNase block (Thermo Fisher) was also prepared and mixed with a single-stranded RNA probe to prevent probe degradation. Hybridization captures were performed on an ABI 2720 PCR machine (Thermo Fisher). The hybridization mixture was covered and incubated overnight (8–16 h) at 65 °C. After the hybridization was complete, the resulting DNA–RNA hybrids were bound to the magnetic beads (Dynabeads MyOne Streptavidin T1, Thermo Fisher). To remove non-specific binding, the magnetic bead–DNA–RNA complex was washed with TargetSeq washing solution (iGeneTech). Finally, PCR enrichment was performed on an ABI 2720 PCR machine (Thermo Fisher) for the target region obtained by hybridization, with parameters as follow: 95 °C for 4 min, 98 °C for 20 s, 65 °C for 30 s, 16 cycles, 72 °C for 30 s, 72 °C for 5 min, and 12 °C hold. The PCR amplification reagent was commercially purchased from KAPA Biosystems. The Nextflex primer was synthesized by Invitrogen, China. The amplified target-region capture library was found to be greater than 3 ng/µL, as determined by a Qubit dsDNA HS Assay Kit (Thermo Fisher). The constructed sequencing library was sequenced on a HiSeq X Ten sequencer (Illumina, San Diego, CA, USA) PE150.

### 2.5. Bioinformatics Analysis

Previously published studies [7,8,16] provide complete details; a brief introduction is as follows: The raw data were first filtered using Trimmomatic software [56] to filter the linker sequences (GATCGGAAGAGCACACGTCT and AGATCGGAAGAGCGTCGTGTAGGGAAAGAGTGT) and low-quality sequences. Low-quality sequences were defined as having a base quality value of less than or equal to 20, corresponding to an accuracy of 99%. Bases that did not satisfy the conditions in the sequence were removed, and sequences having a length of less than 40 bp after base filtration were removed. Finally, FastQC software (https://www.bioinformatics.babraham.ac.uk/projects/fastqc/ (accessed on 15 June 2022), version 0.11.9) was used to perform a quality assessment and ensure clean reads of greater than 95%, with a quality value greater than 30. The clean reads were aligned to the human reference genome (hg19, downloaded from UCSC) using BWA-MEM software [57] to generate an aligned BAM file. To improve the accuracy of the final results the effects of PCR, repetitions in the experiments were removed using Samtools [58] and Picard software (http://broadinstitute.github.io/picard/ (accessed on 15 June 2022), version 2.26.5). Then, a GATK (Genome Analysis Toolkit) [59] was used to detect mutations, such as SNP and InDel, in the results of the alignment. Finally, the results of the detected mutations were annotated with ANNOVAR software [60], and the sequencing depth and coverage were also evaluated.

### 2.6. Association Analysis

The correlation analysis of point mutations and gene levels was completed with reference to a previously published study [61]. In short, We first calculated the groups carrying rare variants, including heterozygous or homozygous mutations, referring to the gnomAD database [62] containing large-scale, whole exomes (123,136 samples, including 8624 people of East Asian ethnicity) and whole-genome sequencing samples. Variants with an East Asian (EAS) allele frequency of less than 0.1 are considered rare. Then, Fisher’s exact test was used to calculate the *p* value of each group; the variants with significant differences were defined as characterized by: (1) both the discovery and validation stages were significant (*p* value of less than 0.05), (2) the combined two-stage data were also significant (FDR value less than 0.05 using Fisher’s exact test); (3) the gene-level association was significant as performed using the SKAT-O method [63] (*p* value of less than 0.001). 

### 2.7. Sanger Sequencing

To confirm the four rare variants, PCR was performed using the ABI9700 PCR system (Thermo Fisher), and the PCR product was then sequenced in an ABI 3730 xl sequencer (Thermo Fisher).

### 2.8. Other Analysis

The lm.fit() function in R software (version 4.0.4) was used to perform a linear fitting analysis on the age and the total number of immune-gene variants in each sample. The violinplot() function in the Python (version 3.7.5) package of Seaborn (version 0.11.1) was used to draw the violinplot; the add_stat_annotation() function in the Python package statannot (version 0.2.3) was used for the statistical significance test, and the set parameters were: test = ‘t-test_ind’, text_format = ‘star’, loc = ‘inside’, verbose = 2.

## 3. Results

**Figure 1:**
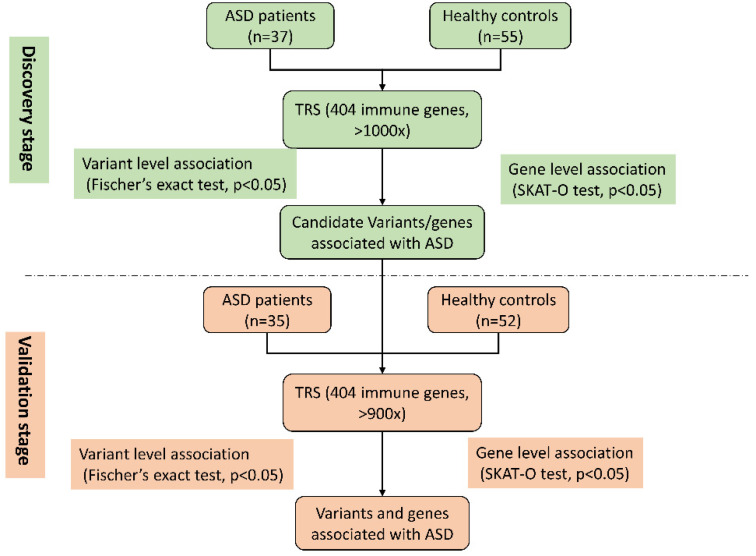
Flowchart of study design.

As shown in Figure 1, the study was conducted in two stages: the discovery stage and the validation stage. In the discovery stage, 37 patients with ASD and 55 healthy controls were recruited. targeted region sequencing was performed using our previously designed panel of 404 immune-response-molecule genes. The target region size was approximately 500 Kbp, the average coverage depth was >1000×, and the 10× coverage rate was >99%. A total of 7526 point mutations were detected, 285 of which differed significantly between the ASD and the healthy control groups (*p* value less than 0.05, Fisher’s exact test, Supplementary Table S1). During the validation stage, 35 patients with ASD and 52 healthy controls were enrolled. Statistical analysis showed that 231 mutations were significantly different between the ASD and healthy control groups (*p* value less than 0.05, Fisher’s exact test, Supplementary Table S2).

Combining these two stages, a total of 61 ASD-related point mutations were found (Supplementary Table S3), 4 of which were rare, functional mutations. These included the missense mutation p.R322H and the splicing mutation c.956-4C>T of the *LILRB2* gene, the missense mutation p.S229N of the *HLA-DQB1* gene, and the missense mutation p.A93G of the *HLA-B* gene (Table 1). These 4 mutations were verified by Sanger sequencing with the primers listed in Supplementary Table S4.

The SKAT-O method, which considers both common and rare variants as risk factors for disease, was used to evaluate the association between the 404 immune-response genes and ASD. The results showed that the correlation *p* value of *HLA-B* and *LILRB2* reached a level of less than 1 × 10^−5^.

Considering that age is an important confounding factor, this controlled study included adults without ASD as controls for individuals with ASD. To assess the potential impact of age on immune-gene-variant counts, we used the lm.fit() function in R software to perform a linear fitting analysis on the age and the total number of immune-gene variants in each sample (Supplementary Table S5). The calculated *p* value was 0.4556, and R-squared was 0.0032 (Supplementary Figure S1), indicating that age effect on the number of immune-gene variants is limited. In ASD individuals, the ratio of male:female was extremely unbalanced, usually 3~4:1, or even higher. In this study, the number of male ASD individuals was three times that of female ASD individuals, which is also typical. Considering that gender is a potential confounding factor for ASD, we used gender as a covariate to evaluate its effect on the four candidate ASD-related immune-gene variants, showing that gender had no effect on these variants (Supplementary Figure S2).

## 4. Discussion

The imbalance of the immune response is considered to play a very important role in ASD. Early studies focused on the discovery of ASD-specific cytokine profiles, lymphocyte subsets, etc. [30,64]; in recent years, researchers have begun to focus on the discovery of maternal autoantibodies [18,21,22,23,25,26] and understanding the role of gut microbiota in it [65,66,67]. Genetic mechanisms are considered to play an important role in ASD, and although a large number of studies have focused on the discovery of ASD-related clinical variants [9,10,11,13,68,69], there is still a lack of systematic evaluation of ASD-related immune-response-gene mutations. In the present study, we systematically evaluated mutations in immune-response molecular genes, including HLA region genes and cytokine and receptor genes, in patients with ASD. Maternal autoantibodies are potential biomarkers for ASD [21,24]; the exposure to maternal autoantibodies during pregnancy is closely related to the ASD-like behavior of the offspring [26,27]. Targeted intervention for ASD patients with positive maternal autoantibodies is a potential treatment for ASD [20]. The HLA system plays an important role in the recognition of autoantibodies; recent studies have found that the HLA system is involved in the regulation of neural development and neuroplasticity, especially through microglia regulation and synaptic pruning [15]. HLA Gene variants are associated with neurodevelopmental diseases such as ASD [70,71]. We found that the missense mutation p.A93G of the *HLA-B* gene from HLA Class I is a risk factor for ASD, which supports the previously published findings that HLA-B*07 alleles are more common in patients with ASD [72] and that *HLA-B* gene polymorphism is related to ASD [73]. We also found that the frequency of the missense mutation p.S229N of *HLA-DQB1* gene in ASD patients (15/72 = 20.83%) was significantly higher than that in healthy controls (4/107 = 3.73%); it is well known that *HLA-DQB1* is a susceptibility gene for celiac disease [74]. Celiac disease is an immune enteropathy, mainly manifested by gluten intolerance, and the symptoms of celiac disease are similar to the gastrointestinal dysfunction prevalent in ASD [75]. Although there is no scientific evidence, the method of intervening in celiac disease using a glutamate-free/casein-free diet is widely used in ASD, and 20–29% of parents report significant improvement in ASD symptoms [76]. We found that 20.83% of patients with ASD had a mutation in a genetic sequence associated with celiac disease risk, which may provide theoretical support for gluten-free/casein-free (GFCF) dietary interventions in patients with ASD; meanwhile, given the high prevalence of gastrointestinal problems in ASD [77], the identified associated variants in *HLA-DQB1* may provide a more prevalent gastrointestinal phenotype than specifically gluten sensitivity in the face of individuals with ASD and gastrointestinal problems. Furthermore, we found that the 2 closely linked mutations, p.R322H and c.956-4C>T, from the *LILRB2* gene had a mutation frequency of 20.83% (15/72) in the ASD patient group, and that these 2 mutations were rare in the control group (2/107 = 1.87%). The *LILRB2* gene is an inhibitory receptor gene of HLA Class I, and it was found that the *LILRB2* gene encodes a neuronal cell-surface receptor that is associated with occurrences of Alzheimer disease as a receptor for β-amyloid [78]. The inhibition of the binding of β-amyloid to LilrB2 has become a potential pathway for the treatment of Alzheimer disease [79]. The results of this study suggest that the *LILRB2* gene may be a potential target for ASD therapy.

The 4 immune-response-gene mutations found in this study were present in 44.44% (32/72) of the ASD patient group and are expected to serve as new targets for the clinical genetic evaluation of ASD. The clinical genetic evaluation of ASD is usually performed using a chromosome microarray combined with whole exome/genome sequencing, which is costly, difficult for data interpretation, and has limited use for clinical genetic evaluation in patients with ASD. The combined mutation frequency of the 4 mutations in ASD patients was 44.44%, with a single mutation frequency of 20.83%. Whole-exome sequencing, whole-genome sequencing, and robust bioinformatic analysis methods have successfully identified high-confidence ASD candidate genes from a large number of de novo and inherited variants [7,8,9,10,11,13,80]. However, despite great efforts, the genetic structure interpretation of ASD using these candidate genes is still incomplete; high-throughput sequencing of immune response genes is expected to complement the ASD genetic machinery.

To our knowledge, this is the first study to screen mutations in immune-response-molecule genes in patients with ASD. This approach can prove helpful in answering whether ASD immune dysfunction has a certain genetic basis. However, this study has certain limitations. First, the genes of the immune-response molecules that we were concerned with may not be comprehensive. This current study only covered cytokines and receptors, HLA regions, and other genes. Second, although we have completely covered the HLA gene-coding region, it does not cover the entire HLA region, and the haplotype of the HLA region could not be obtained. Future studies are planned to evaluate more genes of the immune-response molecule and to perform HLA haplotype detection. Finally, as an exploratory ASD genetic research project, we did not collect detailed psychological or behavioral assessment parameters when we included ASD individuals; the main reason was that we included ASD individuals from several different institutions, and the criteria used for detailed psychological and behavioral assessment of ASD vary from institution to institution. In the future, we will further evaluate the association of immune-gene mutations with psychological and behavioral assessment parameters.

In conclusion, our results suggest that immune dysfunction in patients with ASD may have a genetic basis. The identification of these genetic mutations may also facilitate the development of new genetic, clinical ASD assessment tools. 

## Figures and Tables

**Table 1:** Rare functional mutations in immune-response-molecule genes associated with ASD.

Gene	Variants	“Risk” Allele Carriers		Variant Level *p* Value			FDR	or	SKAT-O Gene-Level *p* Value
		ASD	HC	Discovery	Validation	Combine			
*HLA-B*	p.A93G	11/72	0/107	3.26 × 10^−^^3^	8.79 × 10^−3^	2.73 × 10^−5^	4.48 × 10^−3^	Inf	3.05 × 10^−6^
*LILRB2*	p.R322H	15/72	2/107	4.15 × 10^−4^	2.71 × 10^−2^	2.75 × 10^−5^	4.48 × 10^−3^	13.63	1.53 × 10^−8^
*LILRB2*	c.956-4C>T	15/72	2/107	4.15 × 10^−4^	2.71 × 10^−2^	2.75 × 10^−5^	4.48 × 10^−3^	13.63	1.53 × 10^−8^
*HLA-DQB1*	p.S229N	15/72	4/107	3.16 × 10^−2^	3.21 × 10^−3^	3.81 × 10^−4^	3.95 × 10^−2^	6.70	2.30 × 10^−3^

## Data Availability

Considering the control of human genetic resources, all of the original sequencing data have not been uploaded to the public database. Readers can ask the corresponding author for the variant summary table of the samples.

## Supplementary Materials

The following supporting information can be downloaded at: https://www.mdpi.com/article/10.3390/genes13061098/s1: Supplementary Table S1. Significantly different point mutations identified in the discovery stage; Supplementary Table S2. Significantly different point mutations identified in the validation stage; Supplementary Table S3. Significantly different point mutations identified in the discovery and validation stages; Supplementary Table S4. Primers for four rare variants; and Supplementary Table S5. Metadata and immune-gene variant counts. Supplementary Figure S1. The effect of age on immune gene variant counts; Supplementary Figure S2. The effect of sex on ASD-related immune gene variants.
