# Supplementary material for: Identifying Rare Genetic Variants of Immune Mediators as Risk Factors for Autism Spectrum Disorder
Source: Genes (Basel). 2022 Jun 20;13(6):1098. doi: 10.3390/genes13061098 (PMC9223212; doi:10.3390/genes13061098)
Supplement: Supplementary file 1 [file genes-13-01098-s001.zip › Supplementary_Appendix.pdf]

Supplementary Appendix

Supplementary Figures

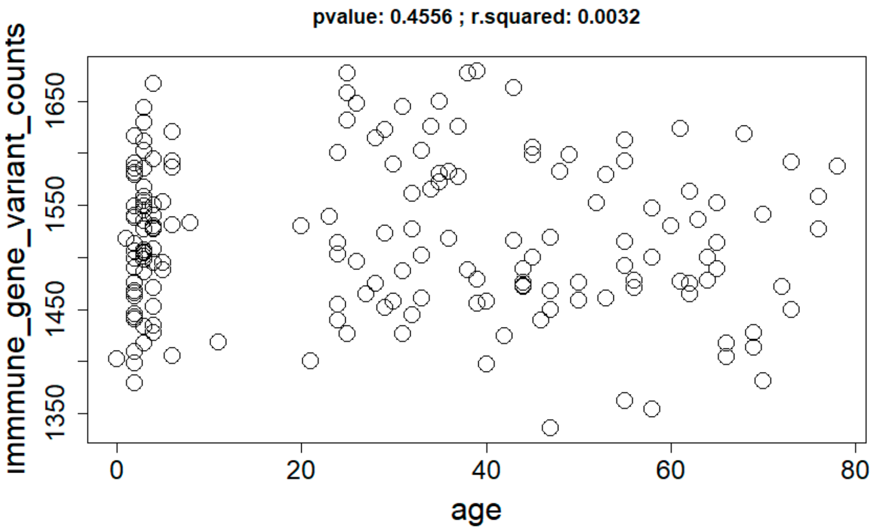

Supplementary Figure S1. The effect of age on immune gene variant counts. The lm.fit() function in the R software was used to perform a linear fitting analysis on the age and the total number of immune gene variants in each sample. The calculated p value was 0.4556 and R-squared was 0.0032, indicating that age effect on the number of immune gene variants is limited.

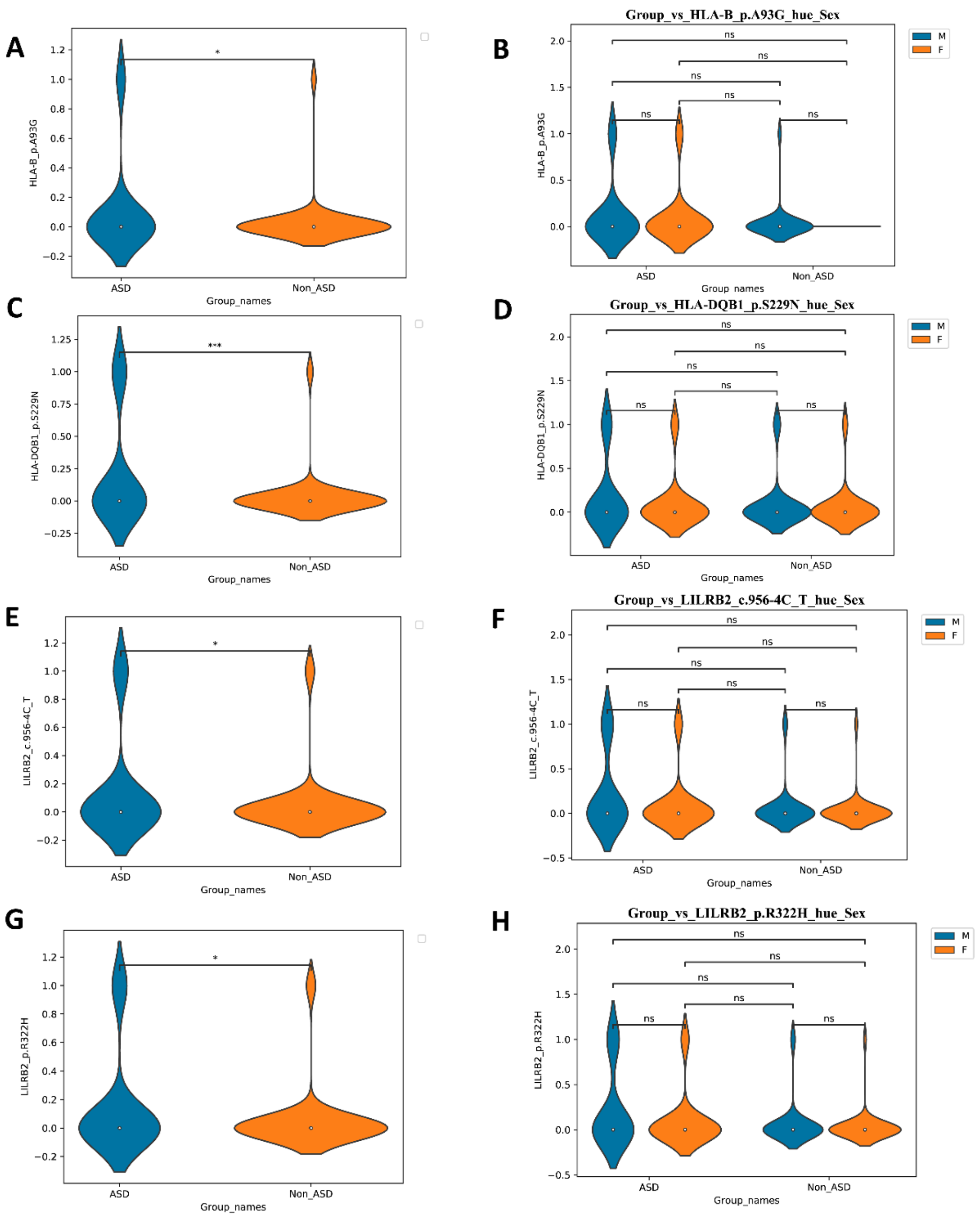

Supplementary Figure S2. The effect of sex on ASD-related immune gene variants. Considering that gender is a potential confounding factor for ASD, gender was used as a covariate to evaluate its effect on the four candidate ASD-related immune gene variants, showing that gender had no effect on these variants. **A**, group_vs_HLA-B_p.A93G; **B**, Group_vs_HLA-B_p.A93G_hue_Sex; **C**, group_vs_HLA-DQB1_p.S229N; **D**, Group_vs_HLA-DQB1_p.S229N_hue_Sex; **E**, group_vs_LILRB2_c.956-4C_T; **F**, Group_vs_LILRB2_c.956-4C_T_hue_Sex; **G**, group_vs_LILRB2_p.R322H; **H**, Group_vs_LILRB2_p.R322H_hue_Sex.
